# Sclerenchymatous ring as a barrier to phloem feeding by Asian citrus psyllid: Evidence from electrical penetration graph and visualization of stylet pathways

**DOI:** 10.1371/journal.pone.0173520

**Published:** 2017-03-09

**Authors:** Justin George, El-Desouky Ammar, David G. Hall, Stephen L. Lapointe

**Affiliations:** 1 USDA-ARS, Subtropical Insects and Horticultural Research Unit, United States Horticultural Research Laboratory, Fort Pierce, Florida, United States of America; 2 University of Florida, IFAS, Lake Alfred, Florida, United States of America; Agricultural Research Organization Volcani Center, ISRAEL

## Abstract

Asian citrus psyllid (*Diaphorina citri*) feeding behaviors play a significant role in the transmission of the phloem-limited *Candidatus* Liberibacter asiaticus (CLas) bacterium that causes the economically devastating citrus greening disease. Sustained phloem ingestion by *D*. *citri* on CLas infected plants is required for pathogen acquisition and transmission. Recent studies have shown a fibrous ring of thick-walled sclerenchyma around the phloem in mature, fully expanded citrus leaves that is more prominent on the abaxial compared with the adaxial side. The composition and thickness of this fibrous ring may have an important role in selection of feeding sites by *D*. *citri* based on leaf age and leaf surface, which in turn can affect pathogen acquisition and transmission. We measured feeding behavior using electrical penetration graph (EPG) recordings of individual *D*. *citri* adults placed on abaxial or adaxial surfaces of young or mature Valencia orange leaves to study the role of the sclerenchymatous ring in modifying *D*. *citri* feeding behavior. Feeding sites on the same leaf tissues were then sectioned and examined by epifluorescence microscopy. The duration of phloem ingestion (E2 waveform) by psyllids was significantly reduced on mature compared with young leaves, and on abaxial compared with adaxial leaf surfaces. The longest duration of phloem ingestion was observed from psyllids placed on the adaxial side of young leaves that had the least developed sclerenchyma. Bouts of phloem salivation (E1 waveform), however, were significantly longer on mature leaves compared with young leaves. *D*. *citri* adults made consecutive phloem feeding attempts (bouts) on the abaxial side of mature leaves and those bouts resulted in unsuccessful or shorter periods of phloem ingestion. Adults also made more frequent and longer bouts of xylem ingestion on mature leaves compared with adult psyllids placed on young leaves. Epifluorescence microscopy showed that the fibrous ring in young leaves was thinner and autofluoresced in red whereas the ring in mature leaves was thicker and autofluoresced in blue, indicating changes in structure and composition (e.g., lignification) of sclerenchyma correlated with leaf age. Our results support the hypothesis that the presence of a thick, well-developed fibrous ring around phloem tissues of mature leaves acts as a barrier to frequent or prolonged phloem ingestion by *D*. *citri* from citrus leaves. This may have an important role in limiting or preventing CLas acquisition and/or transmission by *D*. *citri*, and could be used for identification and development of resistant citrus cultivars.

## Introduction

The Asian citrus psyllid *Diaphorina citri* Kuwayama (Hemiptera: Liviidae) is the primary vector of the phloem-limited bacteria *Candidatus* Liberibacter asiaticus (CLas), putative causal agent of huanglongbing (HLB, or citrus greening disease), currently the world’s most serious disease of citrus [1,2,3]. *D*. *citri* and HLB were first discovered in Asia, but one or both are currently distributed in the Southern region of the United States, the Caribbean, Mexico, Central and South America, Africa, and several countries in South Asia, the Middle East, Reunion and Mauritius islands [1,2,3]. CLas is transmitted in a persistent, propagative manner by both *D*. *citri* nymphs and adults, but nymphs are much more efficient than adults in the acquisition of CLas from infected citrus plants [4,5,6,7]. Early instars of *D*. *citri* feed and grow only on young as opposed to mature citrus leaves and shoots, whereas adults feed on both young and mature leaves. Adult females oviposit exclusively on younger citrus tissues [3].

The Asian citrus pysllid is a phloem specialist that preferentially feeds and reproduces exclusively on flush shoots of rutaceous host plants. Immature *D*. *citri* are only present during the discrete flush cycles of citrus trees. Increases in psyllid densities are strongly related to the presence of new flush shoots [8]. Studies have shown that the availability of flush shoots strongly regulates *D*. *citri* populations in citrus trees, within and between orchard blocks [9, 10], with immature development occurring solely on young shoots [11]. The preference of *D*. *citri* for young expanding shoots could be related to the higher nutritional quality of these shoots and/or to their ease of probing by the insect's mouthparts relative to the lignified tissues of more mature leaves. A recent study by Sétamou et al. [12] showed that leaf nutrient content and amino acid concentration in the phloem of citrus flush shoots affect *D*. *citri* life stages. Young leaves had higher concentrations of macro and micro nutrients relative to mature leaves and this was associated with higher densities of all *D*. *citri* life stages. Concentrations of total and essential amino acids were highest in phloem sap of young expanding flush shoots in both grapefruit and lemon, but dramatically declined as flush shoots matured [12].

Studying the feeding behavior of *D*. *citri* on citrus leaves, especially in relation to phloem feeding activities, is important for understanding the acquisition and transmission of CLas pathogen by the psyllid vector. In previous studies using electrical penetration graphs (EPG), *D*. *citri* adults were found to ingest mainly from phloem sieve elements, although occasionally they also appeared to ingest from xylem [13,14]. However, it was shown that acquisition of CLas from infected plants occurred only when psyllids engaged in sustained phloem ingestion of ≥1 h (behavior corresponding to the E2 waveform) on infected plants [13]. Most (80–90%) of salivary sheath termini produced by *D*. *citri* nymphs and adults that reached a vascular bundle were associated with the phloem, whereas 10–20% were associated with xylem vessels [15]. A fibrous ring of thick-walled sclerenchyma fibers around the phloem in citrus leaves was shown to be more prominent (thicker, with more layers) in fully expanded mature leaves compared with younger leaves, especially on the lower (abaxial) side compared with the adaxial side, and in the midrib compared with secondary veins [15]. In a subsequent study [16], the fibrous ring in the midrib was found to be significantly thicker in one citrus accession (UN-3881) that is relatively resistant to *D*. *citri* adults compared with a susceptible accession (Troyer-1459). Furthermore, fewer *D*. *citri* stylet sheaths were found in the midrib and fewer stylet sheath termini reached the vascular bundle (phloem and/or xylem) of UN-3881 compared with Troyer-1459 plants [16]. These data suggested that the fibrous ring may be a barrier to successful stylet penetration of the vascular bundle and to feeding/ingestion from phloem [13,16].

Here, we used EPG and epifluorescence microscopy to investigate the role of the fibrous ring in young and mature citrus leaves as a barrier to feeding on various tissues by *D*. *citri* adults. We report significant differences in the frequency and duration of waveforms associated with phloem and xylem feeding by *D*. *citri* adults on mature and young leaves, and compare feeding behavior on abaxial and adaxial leaf surfaces. These differences strongly suggest a significant role for the fibrous ring in the feeding behavior of *D*. *citri* on citrus leaves that may be valuable in understanding various aspects of CLas vector relations, e.g., acquisition and inoculation of CLas by *D*. *citri* on young or mature citrus leaves. Insight gained from these studies can also contribute to devising new methods for development of citrus germplasm resistant to *D*. *citri* and/or CLas.

## Materials and methods

### Asian citrus psyllids

Adults psyllids were obtained from a colony established in 2000 at the USDA-ARS U.S. Horticultural Research Laboratory, Fort Pierce, FL. The psyllids were originally collected from citrus in the field and subsequently reared in a greenhouse in cages containing orange jasmine, *Murraya exotica* L. (*Murraya paniculata* auct. non.), until March 2010, when *Citrus macrophylla* Wester was substituted as the rearing plant. The colony is maintained by transferring adults to new plants every 14 d using procedures similar to those described by Skelley and Hoy [17], with no infusion of wild types. The colony is confirmed quarterly by qPCR [18] to be free of CLas. All psyllids used in the study were 8 to 10-day-old adults.

*Plants*: EPG studies were performed on young and mature (fully expanded) leaves of sweet orange seedlings, *Citrus sinensis* Osbeck (cv. Valencia), a popular commercial citrus cultivar. Citrus plants in 3.8 L pots were pruned to initiate flush and were grown under greenhouse conditions. Selected plants were washed and watered 24 h prior to the EPG experiment. Young leaves (soft, immature, lime green, fully expanded ca. 5 cm long and 3 cm wide) and mature leaves (firm, fully expanded, dark green ca. 8.0 cm long and 4.5 cm wide) (S1 Fig) were selected for EPG recordings and for subsequent histological examination. Psyllids were confined to feed on the abaxial (lower) or the adaxial (upper) leaf surface. To facilitate localization of salivary sheaths, psyllid feeding was restricted to a 1.5 cm^2^ area surrounding the midrib of the young or mature leaves. The remaining leaf area was covered with blue labelling tape (Fisherbrand, USA). The length of the gold wire tether was adjusted to confine psyllid movement and feeding to the 1.5 cm^2^ area.

### Electrical penetration graph recordings of *D*. *citri* feeding behaviors

EPG recordings were obtained using a DC-monitor, GIGA-8 model (EPG-Systems, Wageningen, the Netherlands) [19], adjusted to 50x gain. The analog signal was digitized through a DI-710 board, and displayed using Windaq Lite ver. 2.40 software (Dataq Instruments Inc. Akron, OH, USA) on a Dell desktop computer. The EPG monitoring system was housed in a grounded Faraday cage in an environmentally controlled room under continuous lighted conditions. Temperature was set to 26°C with 60–65% RH. Adult *D*. *citri* were collected 4 h prior to the start of the experiment each day and were starved inside collection vials. Psyllids were placed in freezer (-4°C) to immobilize them for 45 s and then held by a plastic pipette tip connected to a gentle vacuum supply under a dissecting microscope. Psyllids were attached to a 1.5 cm long 25 μm-diam. gold wire (Sigmund Cohn Corp., Mt. Vernon, NY) by a droplet of silver conducting paint (Ladd Research Industries, Burlington, VT) applied to the pronotum. The other end of gold wire lead was attached to a copper electrode (3 cm x 1 mm diameter) connected to the EPG probe. The tethered insect was placed on the abaxial or adaxial surface of a young or mature Valencia leaf. To complete the electrical circuit, a reference copper electrode (10 cm x 2 mm) was inserted into the soil medium at the base of the citrus plant. The feeding behaviors of eight individual *D*. *citri* adults (1 male and 1 female for each treatment) on Valencia plants was monitored for 21 hours. EPG recordings were replicated with a total of 10 adults recorded on each of the adaxial and abaxial surfaces of young and mature leaves for a total of 40 individual psyllids and recordings. At the end of each EPG run, the feeding position of the psyllid was marked for sectioning to correlate recorded feeding behavior with the occurrence and pathway of salivary sheath in the leaf tissues.

**Fig 1:**
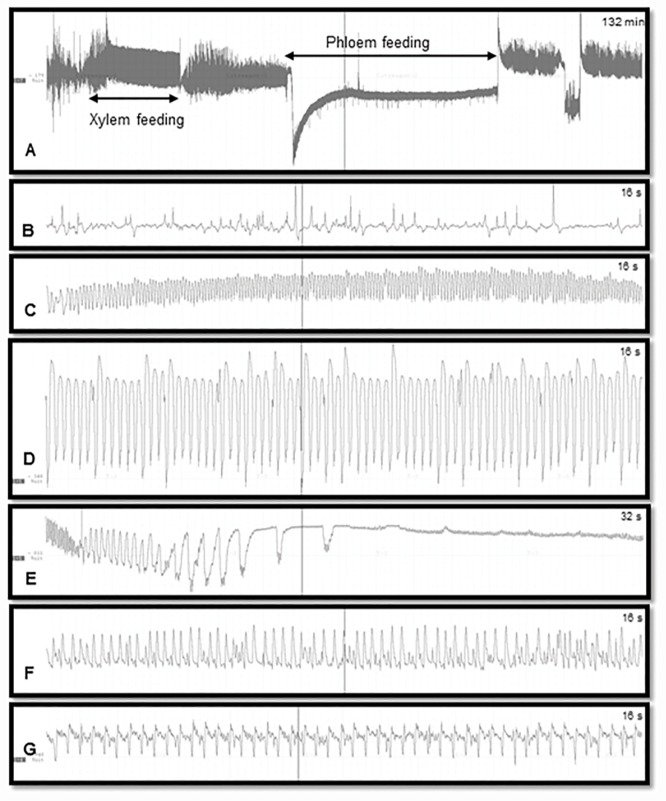
EPG waveforms produced by *D*. *citri* on Valencia orange leaves. A: Xylem and phloem feeding; B: Non-probing (waveform Np); C: Stylet pathway through mesophyll tissues (waveform C); D: Phloem penetration (waveform D); E: Phloem salivation (waveform E1); F: Phloem ingestion (waveform E2); G: Xylem feeding (waveform G).

Characterization of EPG waveforms was accomplished by visually identifying and annotating waveform patterns based on comparison to prior histological studies [13]. Windows Dataq waveform browser (Dataq Instruments Inc., Akron, OH) was used to annotate waveforms. The number and duration of waveform bouts were tabulated in an electronic spreadsheet. The waveforms were visually inspected for frequency patterns and annotated as non-probing (Np), intercellular probing (C), phloem penetration (D), phloem salivation (E1), phloem ingestion (E2) or xylem ingestion (G) (Fig 1). Data for both sexes were pooled, as no significant difference in probing and feeding behavior was observed between male and female adults. Earlier studies also reported no significant differences in probing and feeding behavior of male and female *D*. *citri* with respect to leaf maturity [20]. The waveform associated with phloem feeding activities was carefully analyzed for consecutive or recurring patterns that characterize the presence of sclerenchymatous fibrous ring. Statistical analysis was performed using JMP (v. 10, SAS Inc, Cary, NC). A 2 x 2 factorial design was used to evaluate main effects and interaction of leaf age (young or mature) and leaf surface (adaxial or abaxial) on the number of bouts corresponding to described waveforms and the total duration of each waveform during each 21 h recording.

### Epifluorescence microscopy of leaf tissues and salivary sheaths

Following EPG on each insect, the 1.5 cm^2^ area of the leaf that was exposed to a *D*. *citri* adult during the EPG monitoring period (21 h) was cut from the leaf with a clean razor blade. This area was further divided into smaller pieces about 2 mm wide and 7–8 mm long, and placed in a small microcentrifuge tube with a fixative (4% paraformaldehyde in phosphate buffered saline, PBS), overnight. After washing 3 times in PBST (PBS + 0.1% TritonX 100), each leaf piece was placed in a drop of PBS on a microscope slide and sectioned by hand using a sharp (double edge) razor blade to the thinnest possible sections under a stereomicroscope (at 20X or higher). These sections, determined by confocal microscopy to be ca. 50–70 μm thick [15], were transferred gently (without staining) using fine forceps to a drop of Fluoro-Gel mounting medium (Electron Microscopy Sciences, Hatfield, PA, USA) on another microscope slide.

The autofluorescence of the salivary sheaths and surrounding leaf tissues [15] was examined under UV light using an epifluorescence inverted microscope (Olympus IX70, with 4X or 10X objectives) fitted with a camera and an imaging program (CellSens software, Olympus, Tokyo, Japan). In some cases, low level transmitted light was also used in addition to UV light to help visualize cell boundaries. The occurrence, branching and position where the sheaths terminated (= termini) were recorded in each case. Salivary sheaths were found in some, but not all, of the sectioned leaf pieces. Additional salivary sheaths may have been destroyed or lost in the process of sectioning. The midrib diameter and fibrous ring thickness (width) in both young and mature leaves were measured in images of cross sections (20 sections in each case), using ImageJ program. The fibrous ring was measured around the center on the lower (abaxial) leaf side.

## Results

### Electrical penetration graph recordings of *D*. *citri* feeding behaviors

**Fig 2:**
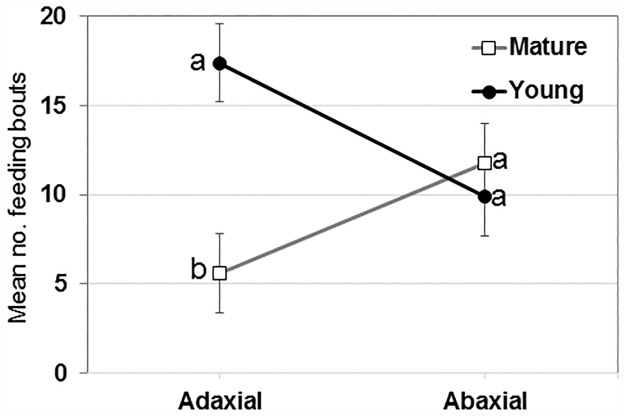
Interaction plot of the number of bouts of G waveforms produced during 21 h recordings by *D*. *citri* adults on adaxial or abaxial leaf surface of young or mature citrus leaves. Means (± SEM, *n* = 10) with the same letters within leaf surface are not significantly different by ANOVA (α = 0.05).

Waveforms produced by *D*. *citri* adults during the 21 h recording periods (Fig 1) were typical for psyllids and readily classified according to the work of Bonani et al. [13], to the following symbols: non-probing (Np), intercellular probing (C), phloem penetration (D), phloem salivation (E1), phloem ingestion (E2) or xylem ingestion (G). There were no significant differences between young or mature leaves or between adaxial and abaxial leaf surfaces in the number of bouts of C, D, E1, E2 and Np waveforms (Table 1). For xylem feeding (waveform G), however, there was a significant difference (*P* = 0.03) due to leaf age and a significant interaction (*P* = 0.004) between leaf age and leaf surface (Fig 2, Table 1). There were significantly more bouts of xylem ingestion (17.4 ± 2.2, *n* = 10) by *D*. *citri* placed on the adaxial (upper) leaf surface of young leaves compared with the number of bouts by psyllids placed on the adaxial surface of mature leaves (6.0 ± 2.2, *n* = 10). An equivalent number of bouts of G waveform was recorded for psyllids placed on the abaxial surface of young (9.9 ± 2.2, *n* = 10) or mature (11.8 ± 2.2, *n* = 10) leaves.

**Table 1:** Main effects of leaf age, leaf surface and their interaction on the number of waveform bouts by *Diaphorina citri* during 21-h. recordings on Valencia orange leaves. Values are mean number of bouts (± SEM, *n* = 20)

Wave-form	Effect	Leaf Age		Leaf Surface		*Pr>F*
		Mature	Young	Abaxial	Adaxial	
C	Age	37 ± 4	38 ± 4			0.89
	Surface			40 ± 4	34 ± 4	0.32
	Interaction					0.68
D	Age	6 ± 1	4 ± 1			0.26
	Surface			6 ± 1	4 ± 1	0.40
	Interaction					0.46
E1	Age	6 ± 1	4 ± 1			0.25
	Surface			6 ± 1	4 ± 1	0.37
	Interaction					0.49
E2	Age	3 ± 1	3 ± 1			0.90
	Surface			3 ± 1	3 ± 1	0.49
	Interaction					0.19
G	Age	9 ± 2	14 ± 2			**0.03**
	Surface			11 ± 2	12 ± 2	0.77
	Interaction					**0.004**
Np	Age	23 ± 3	20 ± 3			0.65
	Surface			25 ± 3	19 ± 3	0.24
	Interaction					0.54

None of the interaction terms for duration of waveforms was significant (α = 0.05, ANOVA). Individual bouts of intercellular probing (waveform C), phloem salivation (E1) and xylem ingestion (G) were significantly longer on mature compared with young leaves (Table 2). Mean duration (± SEM, *n* = 20) of bouts of phloem ingestion (E2) were more than twice as long on young leaves (123 ± 18 min) compared with mature leaves (52 ± 18 min). For phloem ingestion, both main effects were significant (ANOVA, α = 0.05); the leaf age x leaf surface interaction term was not significant (*P>F* = 0.11). E2 bouts by psyllids placed on the adaxial leaf surface were also longer (127 ± 18 min) compared with psyllids placed on abaxial leaf surfaces (49 ± 18) (Table 2).

**Table 2:** Main effects of leaf age and leaf surface, and age x surface interaction on the duration of waveforms produced by *Diaphorina citri* during 21-h recordings on Valencia orange leaves. Values are means in minutes (± SEM, *n* = 20)

Wave-form	Effect	Leaf Age		Leaf Surface		*Pr>F*
		Mature	Young	Abaxial	Adaxial	
C	Age	16.0 ± 0.9	7.3 ± 0.9			**0.03**
	Surface			8.8 ± 0.9	8.6 ± 0.9	0.89
	Interaction					0.94
D	Age	0.8 ± 0.1	0.6 ± 0.1			0.33
	Surface			0.7 ± 0.1	0.7 ± 0.1	0.66
	Interaction					0.54
E1	Age	1.3 ± 0.2	0.6 ± 0.2			**0.04**
	Surface			0.7 ± 0.2	1.2 ± 0.2	0.16
	Interaction					0.30
E2	Age	52.4 ± 18.1	123.3 ± 18.1			**0.008**
	Surface			49.2 ± 18.1	126.5 ± 18.1	**0.004**
	Interaction					0.11
G	Age	40.6 ± 5.0	16.1 ± 5.0			**0.001**
	Surface			23.0 ± 5.0	33.7 ± 5.0	0.14
	Interaction					0.32
Np	Age	37.7 ± 7.8	38.6 ± 7.8			0.93
	Surface			31.1 ± 7.8	45.2 ± 7.8	0.21
	Interaction					0.79

### Phloem feeding

**Fig 3:**
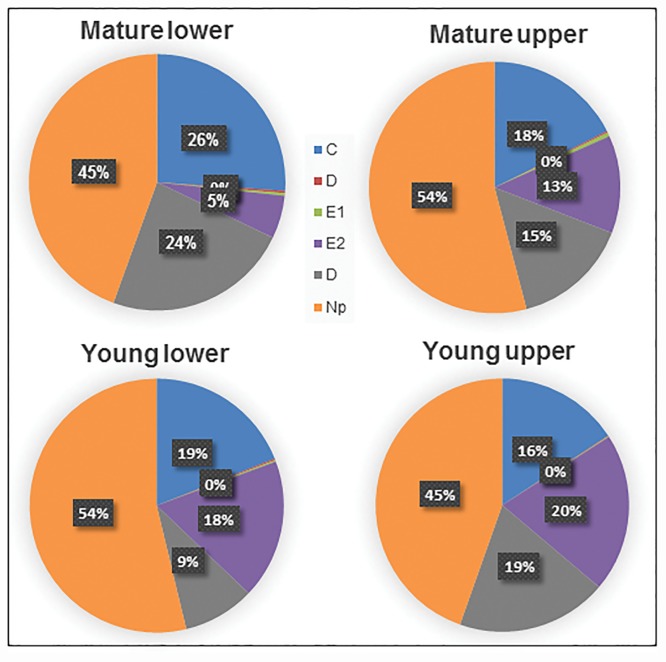
Pie chart showing percentage of the total duration for each feeding activity (waveform) by *D*. *citri* adults on lower and upper surfaces of mature and young leaves. Phloem penetration (D) and salivation (E1) contributed to <1% of total feeding activity. The longest duration of phloem ingestion was observed on young upper leaf surface and shortest phloem ingestion duration on mature lower leaf surface.

**Fig 4:**
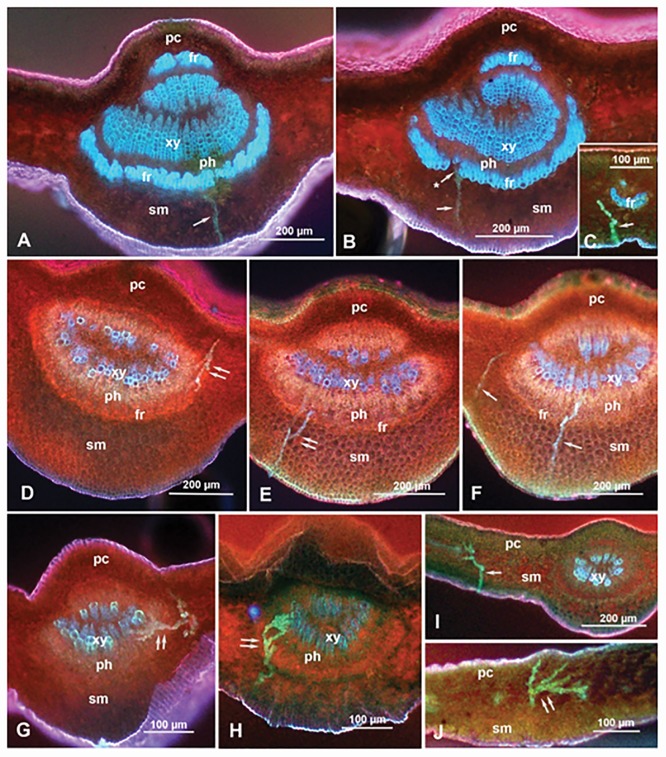
Epifluorescence micrographs showing salivary sheaths (arrows) of *D*. *citri* adults that have been recorded by EPG while feeding on mature (A-C) or young (D-J) citrus leaves, on the adaxial side (D) or the abaxial side (A-C & E-J). Panels C, I and J show sheaths in smaller veins, while the rest of the panels show sheaths in the midrib. Note that the fibrous ring (fr) around the phloem (ph) in the midrib is thicker, and showing more blue color similar to the xylem (xy), in mature leaves (A-C) than in young leaves (D-J). Panel A shows a salivary sheath that apparently terminated very close to fibrous ring. The asterisk in panel B indicates a salivary sheath passing into the phloem through a small gap in fibrous ring. Double arrows indicate branched sheaths (in D, E, G, H & J). Panels E and F show multiple sheaths (representing multiple probes) from the same adult male, while panels G to J show multiple sheaths from an adult female. Low transmitted light (in addition to UV light) was used in panels D-F to show mesophyll cell boundaries. Abbreviations: fr, fibrous ring; pc, palisade cells, ph, phloem; sm, spongy mesophyll; xy, xylem.

Phloem ingestion was initiated by penetration of sieve elements (waveform D) followed by salivation (E1) and ingestion (E2) (Fig 1, Tables 1 and 2). No significant differences were observed for duration (*P* = 0.33) or number of phloem penetration attempts (*P* = 0.26) on young and mature leaves. Psyllids exhibited no differences (*P* = 0.25) in the number of phloem salivation activities following phloem penetration (Tables 1 and 2). A significantly longer duration of phloem salivation, however, was observed on mature compared with young leaves (*F*_*3*, 36_ = 4.13; *P* = 0.04). On the other hand, the duration of phloem ingestion from younger leaves (123.3 ± 18.0 min) was significantly longer than that from mature leaves (52.4 ± 18.0 min) (*P* = 0.008) (Table 2). Also significantly longer phloem ingestion was exhibited on upper leaf surface (132.5 ± 18.0 min) compared to lower surface (49.2 ± 18.0 min) (*P* = 0.004) (Tables 1 and 2). Psyllid adults spent more time ingesting from phloem elements when placed on the adaxial surface of young leaves compared with psyllids placed on either leaf surface of mature leaves. The proportion of time spent engaged in phloem ingestion was longer from the adaxial surface of young leaves (20%) compared with the abaxial surface of mature leaves (5%) (Fig 3). The abaxial surface of mature leaves exhibited the least duration of phloem feeding (34.5 min) compared with young adaxial leaf surface (182.8 min) (Table 2). The significant decrease in duration of phloem ingestion and corresponding increase in phloem feeding attempts in mature leaves may be attributed to the sclerenchymatous fibrous ring, which is most prominent (thicker/more developed) on the lower surface of mature leaves, and least developed in the upper surface of younger leaves (Fig 4A–4I). Adult *D*. *citri* conducted fewer phloem feeding bouts on the adaxial side of young leaves, which resulted in prolonged phloem ingestion. In the case of young leaves, 82% (lower surface) and 88% (upper surface) of phloem salivations (E1) ended in successful phloem feeding (E2). For mature leaves, only 42% (lower) and 55% (upper) of phloem salivation (E1) activities resulted in successful phloem feeding (E2).

**Fig 5:**
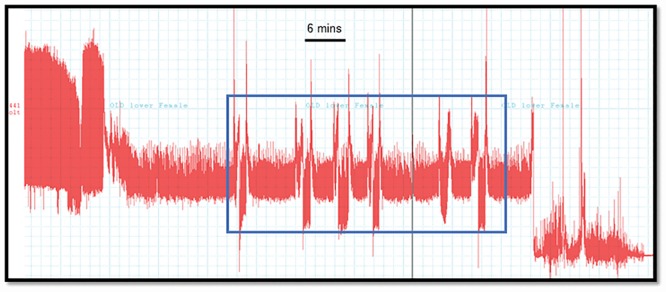
A section of EPG recording of *D*. *citri* feeding on abaxial surface of mature leaves showing consecutive phloem feeding attempts on mature leaves resulting in no or little phloem ingestion (E2). This pattern was observed in 7 of 10 EPG recordings of psyllids placed on the abaxial surface of mature leaves.

We frequently observed a consecutive pattern of waveforms (C-D-E1-C-D-E1) of intercellular stylet penetration (C) phloem penetration (D) phloem salivation (E1) that resulted in unsuccessful or shorter duration of phloem ingestion (E2) from psyllids placed on the lower surface of mature leaves (Fig 5). The duration of phloem salivation was also significantly shorter (*F*_*3*,36_ = 4.1; *P* = 0.04) on younger leaves (39 sec) compared with mature leaves (77 sec) (Table 2, Fig 3). This longer duration of phloem salivation (E1), and decreased phloem ingestion (E2) on mature leaves could be attributed to the presence of a well-developed sclerenchymatous fibrous ring that makes the phloem elements of mature leaves less accessible for feeding by *D*. *citri* and therefore for pathogen acquisition and transmission. The duration of intercellular stylet penetration (waveform C) associated with psyllid feeding behavior was significantly longer in mature compared with young leaves (*F*_*3*,36_ = 5.2; *P* = 0.02). No differences were observed in the number of stylet penetration (C) attempts between leaf surfaces. Also no differences were observed in non-probing (Np) of *D*. *citri* on young or mature leaves.

### Xylem feeding

A significantly higher number of xylem feeding attempts (*F*_*3*,36_ = 4.92; *P* = 0.03) was observed on the young (17.4 ± 2.6) compared to mature upper leaf surface (6.0 ± 1.5) (Fig 2). Also, a significant interaction was observed between leaf age and leaf surface (*F*_*3*,36_ = 9.44; *P* = 0.004); no effect of leaf age on lower leaf surfaces was observed (*P* = 0.77) (Table 1, Fig 2). Significantly longer xylem ingestion bouts were conducted by psyllids placed on mature leaves (40.6 ± 5.0 min) compared with those conducted by psyllids placed on younger leaves (16.1 ± 5.0 min) (*F*_*3*,36_ = 12.2; *P* = 0.001). There was no effect of leaf surface (*P* = 0.13), or interaction of leaf age x leaf surface (*P* = 0.32) on xylem ingestion duration (Table 2). The total duration of xylem ingestion was longest for psyllids placed on the lower surface of mature leaves (24%), and shortest for psyllids placed on the lower surface of young leaves (9%) (Fig 3). The mean duration of individual xylem ingestion bouts was longest for psyllids placed on the upper surface of mature leaves, and young upper and lower surfaces had shortest xylem feeding bouts.

### Epifluorescence microscopy of leaf tissues and salivary sheaths

The histology and autofluorescence of cross sections in the leaf midrib or smaller veins of both young and mature Valencia leaves are shown in Fig 4A–4J. Mature leaves had significantly wider mean (± SEM) midrib diameter (737 ± 26 μm) than young leaves (610 ± 42 μm) (*t* = 5.37, d.f. = 38, *P*<0.0001). The fibrous ring was also thicker (wider) in mature leaves (31 ± 2 μm) compared with that in young leaves (17 ± 1 μm) (*t* = 13.64, df = 38, *P*<0.0001). On individual leaves tested (young and mature), no correlation was found between fibrous ring thickness or midrib diameter and the number of feeding bouts. An inverse correlation between fibrous ring thickness and duration of phloem ingestion was observed but was slightly less than significant (*F*_*1*,*8*_ = 4.82; *P* = 0.059). This suggests that the fibrous ring thickness may not be the only factor affecting the decreased phloem ingestion from mature leaves, and that other factors, e.g., chemical composition, may play a role as well.

Autofluorescence (under UV light) of the fibrous ring and xylem vessels was very different between young and mature leaves. In mature leaves, both the xylem elements and the fibrous ring auto-fluoresced in bright blue (Fig 4A–4C), whereas in younger leaves only the inner xylem vessels (apparently, the mature ones) were blue while the outer xylem and fibrous ring were reddish in color, similar to the walls of mesophyll cells (Fig 4D–4F). Blue autofluorescence is probably correlated with lignification of secondary cell walls in xylem vessels and the fibrous ring during leaf maturation (see Discussion).

Significantly more salivary sheaths were found in young (30 sheaths in 9 leaves) compared with mature leaves (11 in 7 leaves) (*P*<0.05, Table 3), and a greater proportion of sheath termini were found in the vascular bundle of young (63%) compared with mature leaves (18%) (*P*<0.012). Most of these sheaths were found in the midrib (70% in young and 64% in mature leaves) and the rest were found in smaller veins (Fig 4). Some of the salivary sheaths had two or more branches; significantly more branched sheaths were found in young (30%) compared with mature leaves (0%) (*P*<0.042, Fig 4D, 4E & 4G–4J). In young leaves, 94% of sheath termini were found in the phloem, and 59% were found in the xylem; some branched sheaths were found in both (Fig 4D, 4G and 4H). The proportion of sheaths that terminated just before or very close to the fibrous ring (apparently without entering the vascular bundle) was significantly higher in mature leaves (3/11) compared to those in young leaves (0/30) (*P*<0.003). All three sheaths observed in mature leaves were initiated on the abaxial surface of mature leaves (Fig 4A).

**Table 3:** Occurrence and terminal position (terminus) of salivary sheaths produced by *D*. *citri* adults placed on young or mature Valencia leaves for 21 h.

Proportion	No. (ratio)		*χ*^*2*^, d.f. = 1	*P>χ*^*2*^
	Young	Mature		
Leaves with sheaths/leaves examined	9/20 (0.45)	7/22 (0.32)	0.77	0.38
Sheaths found/leaves with sheaths	30/9 (3.33)	11/7 (1.57)	3.83	**0.05**
Sheaths in midrib/all sheaths	21/30 (0.70)	7/11 (0.64)	0.15	0.70
Sheath termini near fibrous ring*	0/30 (0.00)	3/11 (0.27)	8.83	**0.003**
Sheath termini in vascular bundle/sheaths observed	17/27 (0.63)	2/11 (0.18)	6.27	**0.012**
Branched sheaths/sheaths observed	8/27 (0.30)	0/11 (0.00)	4.13	**0.042**
Sheath termini in phloem/termini in vascular bundle	16/17 (0.94)	-	-	
Sheath termini in xylem/termini in vascular bundle	10/17 (0.59)	-	-	

*All termini were produced by psyllids placed on the abaxial leaf surface.

## Discussion

Huanglongbing (citrus greening), caused by CLas transmitted by *D*. *citri*, is the most destructive citrus disease in Florida, Brazil, Central America and other citrus growing areas worldwide. In both Florida and Brazil, the pathogen has spread rapidly throughout commercial and residential citrus plantings causing major losses to the citrus industry [2]. The management program against HLB involves intensive chemical control of *D*. *citri*, removal of HLB-infected trees, and planting of disease-free nursery stock [2,3]. However, intensive use of insecticides against psyllids is expensive, disruptive to beneficial parasitoids and predators, and unsustainable. Intensive chemical control of *D*. *citri* can also be ineffective in preventing the introduction and spread of CLas in new citrus plantings [3]. Thus, development of more effective measures for management of psyllids and HLB, especially resistant genotypes to *D*. *citri* and/or CLas, is of critical importance to ensure the sustainability of commercial citrus in Florida, Brazil and other citrus growing areas where the disease is spreading.

The ‘fibrous ring’ partially surrounding the phloem tissue in the midrib and other veins in citrus leaves is composed of a few layers of compact thick-walled fibers [15,16]. This ring has been referred to previously as sclerenchyma fibers [21], sclerenchymatic sheath [22], phloem fibers [23], undifferentiated pericyclic fibers [13] and fiber strand [24]. Richter et al. [25], studying *Phormium tenax* leaf tissues, observed the highest lignification in the cell corners and cell walls of the sclerenchyma fibers surrounding the vascular tissue, and suggested that this lignification can act as a protective barrier for the vascular tissue. Ammar et al. [15] stated that the fibrous ring in citrus leaves is more prominent (thicker, with more layers and fewer or narrower gaps) in mature than in young leaves and on the abaxial than the adaxial side of the leaf, whereas it is rudimentary or absent from the adaxial side of secondary veins. They suggested that the prominence of the fibrous ring in mature leaves may explain why *D*. *citri* nymphs prefer to settle and feed on young flush rather than mature leaves, and why adults prefer to settle and feed on secondary veins compared with the midrib on both upper and lower leaf surfaces. Here we examined the feeding behavior of *D*. *citri* adults on both sides of young and mature Valencia leaves using both EPG and fluorescence microscopy of sectioned leaf parts where psyllids were feeding during EPG monitoring. The longest duration of phloem ingestion (E2 waveform) was observed on the adaxial surface of young flush leaves that had the least developed sclerenchymatous fibrous ring, whereas the shortest phloem ingestion duration occurred on the abaxial side of mature leaves where this fibrous ring was most developed. Contrary to phloem ingestion, bouts of phloem salivation were significantly longer on mature leaves compared with that on young flush. EPG results also showed that *D*. *citri* adults made consecutive phloem feeding attempts when placed on the abaxial side of mature leaves. Those bouts resulted in unsuccessful or shorter periods of phloem ingestion. We showed for the first time that these unsuccessful bouts in mature leaves were associated with a consecutive pattern of waveforms (C-D-E1-C-D-E1) of intercellular stylet penetration (C) phloem penetration (D) phloem salivation (E1) activities that resulted in unsuccessful or a reduced duration of phloem ingestion. This pattern was consistently observed only on lower surface of older leaves and was absent in waveform patterns of younger flushes. Bonani et al. [13] reported that within 5h, 50% of adult *D*. *citri* were able to ingest phloem from young flush whereas only 15% ingested from phloem when placed on older leaves. Using histological correlations we were able to show that prolonged phloem salivation and decreased phloem ingestion from mature leaves can be attributed to the presence of a well-developed sclerenchymatous fibrous ring.

Interestingly, *D*. *citri* adults made more frequent and longer bouts of xylem ingestion on mature leaves compared with psyllids placed on young leaves, perhaps to compensate for the lack of phloem feeding in old mature leaves. Ammar et al. [16] reported that *D*. *citri* adults seemed to divert their stylets around the fibrous ring to enter the vascular bundle in citrus leaves through gaps at the fibrous ring. Similarly, the aphid *Megoura viciae* (Buckton) (Hemiptera: Aphididae) was reported to make a stylet ‘detour’ when encountering arches of tough sclerenchyma cells along the phloem in their host plant *Vicia fabae* L. [26]. Cen et al. [14], using EPG on psyllids feeding on healthy or CLas-infected citrus plants, showed that the time to first and sustained salivation was longer in infected compared with healthy plants. Also with the progress of infection, the percentage time spent by *D*. *citri* on phloem salivation (E1) increased. Cen et al. [14] hypothesized that the fibrous ring around the phloem, which is thicker in diseased than healthy leaves, may be at least partly responsible for the increased time before the first and sustained salivation in the phloem by *D*. *citri* adults.

Our fluorescence microscopy results showed that a greater proportion of sheath termini were found in the vascular bundle in younger compared with older leaves. We also demonstrated that the fibrous ring in mature leaves is not only significantly thicker in mature leaves, but it also autofluoresced in blue similar to xylem vessels. In younger leaves, however, both the fibrous ring and part of the xylem vessels closer to the phloem autofluoresced in red similar to that of mesophyll cell walls. This indicates that the composition of the fibrous ring, as well as its thickness, are different between young and mature leaves. An inverse correlation between the fibrous ring thickness and the duration of phloem ingestion was slightly less than significant (*P* = 0.059), which suggests that lignification of the fibrous ring in mature leaves may be as important as its thickness in affecting the psyllid’s feeding behavior. Wang et al. [27] reported that the cell walls of both xylem and sclerenchyma are characterized by the presence of the heterogeneous lignin polymer that plays an essential role in their physiology. Lignin content of plant tissues has often been correlated with resistance to insects and plant pathogens. Blum [28] identified lignin as a major factor in resistance of sorghum against *Atherigona variasoccata* Rondani (Diptera: Muscidae). Akin and Hartley [29] indicated that lignin-like aromatics increased in older sclerenchyma tissues, whereas ester- or ether-linked phenolic acids accounted for most of the UV absorption in young sclerenchyma and young and old parenchyma. Rangasamy et al. [30] studied the distribution of salivary sheaths of the chinch bug (Hemiptera: Blissidae) and axillary shoot lignification in resistant and susceptible cultivars of St. Augustine grass *Stenotaphrum secundatum* (Walter). Salivary sheaths were more abundant on the outermost leaf sheath of axillary shoots of resistant cultivars compared with susceptible ones. These results, combined with electron microscopy, suggested that the thick-walled sclerenchyma fibers around the vascular bundle play a role in southern chinch bug resistance in St. Augustine grass, possibly by reducing stylet penetration to the vascular tissue [30]. Sétamou et al. [8] and Hall et al. [31] reported that the transmission rates of CLas by *D*. *citri* were enhanced by the presence and developmental stage of citrus flush. Young plants containing flush were more prone to contract HLB compared with older flush [8,31]. This increased HLB transmission in young flushes could be directly related to *D*. *citri* feeding behavior reported in our study, as more phloem feeding was observed on young leaves compared with mature leaves. Ammar et al. [16] found fewer salivary sheaths in the midrib and fewer sheath termini reached the vascular bundle (phloem and/or xylem) in a *D*. *citri-*resistant genotype (UN-3881) compared with a susceptible one (Troyer-1459). Additionally, they reported that in midribs of UN-3881 leaves, the fibrous ring was significantly wider (thicker) compared with that in midribs of Troyer-1459, which suggested that the thickness of the fibrous ring may be a barrier to stylet penetration to the vascular bundle. These results as well as our current work support the hypothesis that the presence of a well-developed fibrous ring around phloem tissues of mature leaves acts as a barrier to frequent or prolonged phloem ingestion by *D*. *citri* from citrus leaves. This may have an important role in limiting or preventing CLas acquisition and/or transmission by *D*. *citri*, and could be used for identification and development of resistant citrus cultivars. Further work is needed to explore the use of this trait in designing or selecting citrus genotypes that are resistant to psyllids and/or CLas.

## Supporting information

S1 Fig The size and color of young and mature Valencia citrus leaves used in the EPG and histological studies. A) Adaxial surface of mature leaf; B) Abaxial surface of mature leaf; C) Adaxial surface of young leaf; D) Abaxial surface of young leaf.
(TIF)
