# Supplementary figures and images for: Sclerenchymatous ring as a barrier to phloem feeding by Asian citrus psyllid: Evidence from electrical penetration graph and visualization of stylet pathways
Source: PLoS One. 2017 Mar 9;12(3):e0173520. doi: 10.1371/journal.pone.0173520 (PMC5344446; doi:10.1371/journal.pone.0173520)

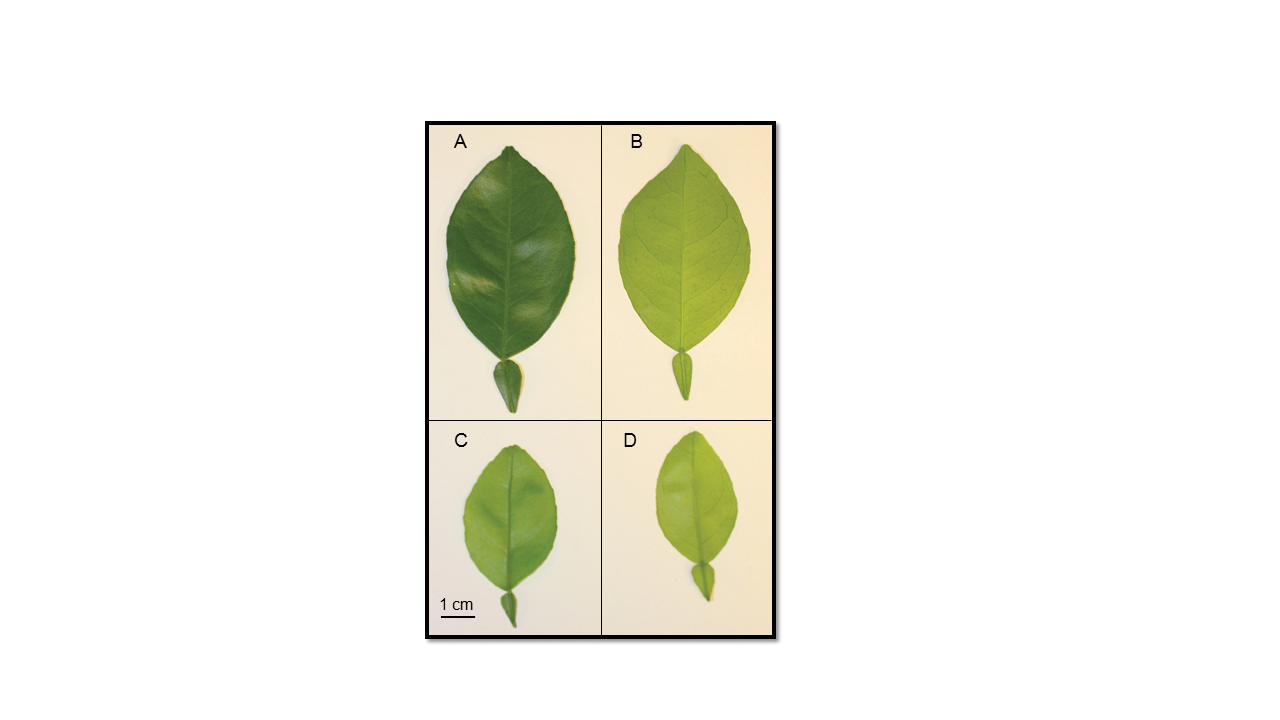

Supplement: S1 Fig — A) Adaxial surface of mature leaf; B) Abaxial surface of mature leaf; C) Adaxial surface of young leaf; D) Abaxial surface of young leaf. (TIF) [file pone.0173520.s001.tif]
